# Validation of Two Short Personality Inventories Using Self-Descriptions in Natural Language and Quantitative Semantics Test Theory

**DOI:** 10.3389/fpsyg.2020.00016

**Published:** 2020-02-19

**Authors:** Danilo Garcia, Patricia Rosenberg, Ali Al Nima, Alexandre Granjard, Kevin M. Cloninger, Sverker Sikström

**Affiliations:** ^1^Blekinge Center of Competence, Karlskrona, Sweden; ^2^Department of Psychology, University of Gothenburg, Gothenburg, Sweden; ^3^Department of Behavioral Science and Learning, Linköping University, Linköping, Sweden; ^4^Anthropedia Foundation, St. Louis, MO, United States; ^5^Department of Psychology, Lund University, Lund, Sweden

**Keywords:** character, identity, quantitative semantic test theory, narrative self, personality

## Abstract

**Background:**

If individual differences are relevant and prominent features of personality, then they are expected to be encoded in natural language, thus manifesting themselves in single words. Recently, the quantification of text data using advanced natural language processing techniques offers innovative opportunities to map people’s own words and narratives to their responses to self-reports. Here, we demonstrate the usefulness of self-descriptions in natural language and what we tentatively call Quantitative Semantic Test Theory (QuSTT) to validate two short inventories that measure character traits.

**Method:**

In Study 1, participants (*N*_1_ = 997) responded to the Short Character Inventory, which measures self-directedness, cooperativeness, and self-transcendence. In Study 2, participants (*N*_2_ = 2373) responded to Short Dark Triad, which measures Machiavellianism, narcissism, and psychopathy. In both studies, respondents were asked to generate 10 self-descriptive words. We used the Latent Semantic Algorithm to quantify the meaning of each trait using the participants’ self-descriptive words. We then used these semantic representations to predict the self-reported scores. In a second set of analyses, we used word-frequency analyses to map the self-descriptive words to each of the participants’ trait scores (i.e., one-dimensional analysis) and character profiles (i.e., three-dimensional analysis).

**Results:**

The semantic representation of each character trait was related to each corresponding self-reported score. However, participants’ self-transcendence and Machiavellianism scores demonstrated similar relationships to all three semantic representations of the character traits in their respective personality model. The one-dimensional analyses showed that, for example, “loving” was indicative of both high cooperativeness and self-transcendence, while “compassionate,” “kind,” and “caring” was unique for individuals high in cooperativeness. The words “kind” and “caring” indicated low levels of Machiavellianism and psychopathy, whereas “shy” or “introvert” indicated low narcissism. We also found specific keywords that unify or that make the individuals in some profiles unique.

**Conclusion:**

Despite being short, both inventories capture individuals’ identity as expected. Nevertheless, our method also points out some shortcomings and overlaps between traits measured with these inventories. We suggest that self-descriptive words can be quantified to validate measures of psychological constructs (e.g., prevalence in self-descriptions or QuSTT) and that this method may complement traditional methods for testing the validity of psychological measures.

## Introduction

Human personality can be defined as the dynamic organization, within the person, of biopsychosocial systems that regulate adaptation to a changing environment (Cloninger et al., 1993; see also Cloninger et al., 2019). This includes systems of self-government that modulate cognitions, emotions, impulse control, and social relationships. In this context, specific personality traits are responsible for how the individual perceives and thinks about oneself, other people, and the world as a whole (Cloninger, 2004, 2009), which are aspects that are strongly associated to physical, mental, social, and spiritual health (Vaillant and Vaillant, 1990; World Health Organization [WHO], 2001; Cloninger, 2003, 2004; VanderWeele, 2017). The measuring of personality is often done using self-reports, something that is not without controversy regarding conceptualization and measure accuracy (cf. Cloninger et al., 2019). For instance, although trait models of personality stem from natural self-descriptive language (Leising et al., 2014), the validation of inventories that measure personality and most psychological constructs is often done using Classical Test Theory (CTT) and more recently using Item Response Theory (IRT) rather than natural language. This is important because individual differences are expected to be encoded in natural language if they are relevant and prominent features of personality, thus, manifesting themselves in single words (cf. the psycholexical hypothesis; John et al., 1988). These single words might be used in self-descriptions, which in turn reflect people’s temperament and own concept of the self or character, including the perception of her/his identity (Adams et al., 2012). In one study, for example, researchers found 624 adjectives that laypeople used when freely generating words to describe people they know (Leising et al., 2014). What is more, the adjectives that these participants rated as more important were found more frequently in an independent large text corpus of 500 million words of online communication. Hence, suggesting that the words people frequently use to describe personality might indeed be valid to describe human temperament and character (cf. Garcia et al., 2015).

Despite the fact that CTT and IRT are good methods for the validation of measures, there are some limitations. For instance, CTT methods are dependent on the number of items and on the sample’s size and other features, so any changes to these features can strongly affect both item and the total psychometric properties of the scale. Moreover, IRT methodology does not address, for example, the issue of social desirability or response style (Oishi, 2007). We argue that using, for example, the words people use to describe themselves might serve as a new tool to validate measures of personality and other psychological phenomena. One obstacle, however, has been that advanced methodological techniques are necessary to actually use freely generated self-descriptive words in such analyses. Researchers have only recently started using these techniques in the social sciences (see Leising et al., 2014; Sikström and Garcia, 2019). Indeed, despite the fact that lexical models of personality have their basis in natural language, self-descriptive words have not been mapped to specific personality constructs to distinguish meaningful patterns that explain people’s behavior and tendencies (for a review, see Uher, 2013). Importantly, at times, researchers look for short measures for the assessment of personality, which might compromise validity. Moreover, regarding personality, different measures can be used that are, for example, stated as representing a dark side of personality rather than just personality. Thus, making psychometric scrutiny regarding these short measures even more important, if we do not want to risk ending up with “quick and dirty measures” that lack a comprehensive theory (cf. Wong and Roy, 2018) and suffer of “jingle-jangle” fallacy^1^ (cf. Kelley, 1927; Block, 1995).

More recently, the quantification of text data using advanced natural language processing techniques offers innovative opportunities to map people’s own words and narratives to their responses to self-reports’ scales. Here, we demonstrate the usefulness of what we tentatively call Quantitative Semantics Test Theory (QuSTT) to validate two short inventories that measure character traits. We use the Latent Semantic Analysis algorithm, which is not only a method but also a theory for how humans acquire, induct, and represent meaning and knowledge (Landauer and Dumais, 1997; Landauer, 2008). By applying this statistical computation on a large text corpus, researchers can extract and represent the meaning of words based on the context in which it co-occurs with other words. We expected that the quantified meaning of words that an individual uses to intentionally describe herself/himself may predict her/his level in different personality traits. We aim to exemplify this by mapping the words that participants use to their responses in each scale and also to personality profiles. Before stating any further expectations, we present the personality models in each study.

### Light Character Traits: Self-Directedness, Cooperativeness, and Self-Transcendence

Cloninger proposed in his model of personality (Cloninger et al., 1993) four dimensions of temperament and three dimensions of character. Here, we focus on character, which can be defined as what the individual makes of her/himself intentionally or individual differences in values, goals, and self-conscious emotions, such as, hope, love, and faith (Cloninger, 2004). We do this partially for practical reasons; the shortest measure derived to measure these dimensions assesses only the three character traits, but also because the light character traits stand in contrast to the Dark Triad traits (Garcia and Rosenberg, 2016). The three character traits are the following: (1) *self-directedness*, which refers to the person’s level of self-determination and tendency to self-control, self-sufficiency, self-acceptance, responsibility, and reliableness; (2) *cooperativeness*, accounts for individual differences in social acceptance, tolerance toward others, and tendency to be a helpful and empathic person; and (3) *self-transcendence*, which refers to the person’s tendency to experience self-forgetfulness, spiritual acceptance, and to be patient and imaginative (Cloninger et al., 1993; Köse, 2003). In this context, Cloninger developed the Temperament and Character Inventory for the assessment of personality according to his biopsychosocial model^2^ (Cloninger et al., 1993; see also Garcia et al., 2017). The original long version comprises 240 items that operationalize the four temperament dimensions and the three character dimensions, while the inventory that we investigate here is a short version that measures the character traits using 15 items (i.e., the Short Character Inventory).

**FIGURE 1:**
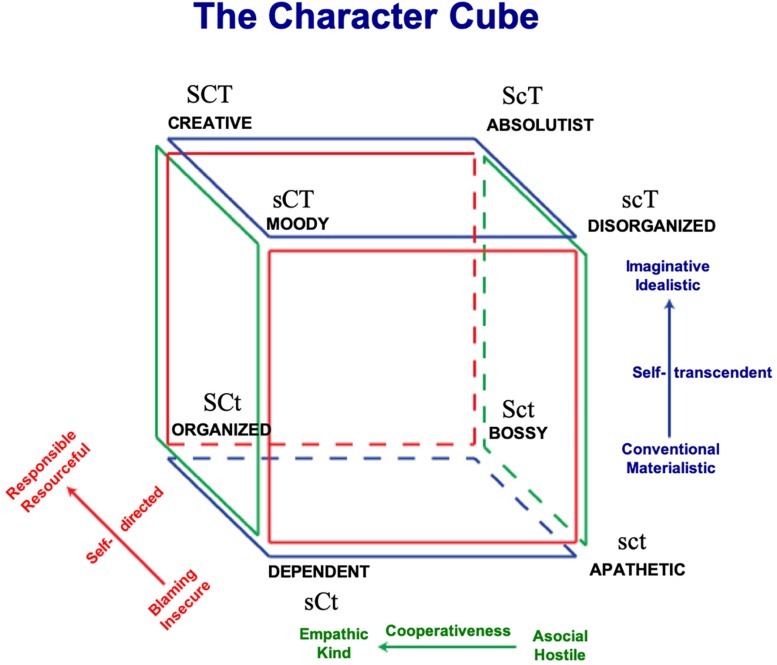
The character cube representing the eight possible combinations of high and low scores in Cloninger’s light character traits. Reprinted with permission from Anthropedia Foundation. S/s, high/low self-directedness; C/c, high/low cooperativeness, T/t, high/low self-transcendence.

As the long version, this short version was designed to be applicable to large normal populations without being stigmatizing or pathologizing. Furthermore, instead of assuming that personality can be decomposed into independent dimensions, Cloninger based his personality model and inventories on complex interactions, such as gene–gene and gene–environment (Cloninger, 2004; Zwir et al., 2018a, b, 2019). Thus, personality is a dynamic complex adaptive system. In other words, on a daily basis a person is adapting not only to the environment but also to the emotions and cognitions within her/himself. This notion of personality as whole system unit has been suggested to be best studied by analyzing “common types” or profiles, see Figure 1 (Bergman and Magnusson, 1997; Cloninger et al., 1997; Bergman and Wångby, 2014; Zwir et al., 2018a, b, 2019). For instance, perceptual aberrations such as superstitious or magical thinking and vulnerability to overvalued ideas or psychosis is a product of excessive imagination (i.e., high self-transcendence) in combination with lack of solid reality testing (i.e., low self-directedness) (Smith et al., 2008). Moreover, individuals who report high levels in all three character traits (i.e., “Creative” profile) or high levels in self-directedness and cooperativeness, but low in self-transcendence (“Organized” profile) report the highest levels of health, well-being, longevity, and functionality (Cloninger, 2004). Creative people are expected to see life as being filled with opportunities to learn from mistakes (i.e., high self-directedness), to work in the service of others (i.e., high cooperativeness), and to grow in awareness (i.e., high self-transcendence) around life as a whole and what is beyond human existence (Cloninger, 2004). In contrast, people with an “Apathetic” profile are low in all three traits of character, so they often think “life is hard, people are mean, and then you just die!” Not surprisingly, they are unhappy, alienated, and physically unhealthy and fearful of death with high rates of mental and physical disorders (Cloninger, 2004) (see Figure 1).

### The Dark Triad: Machiavellianism, Narcissism, and Psychopathy

**FIGURE 2:**
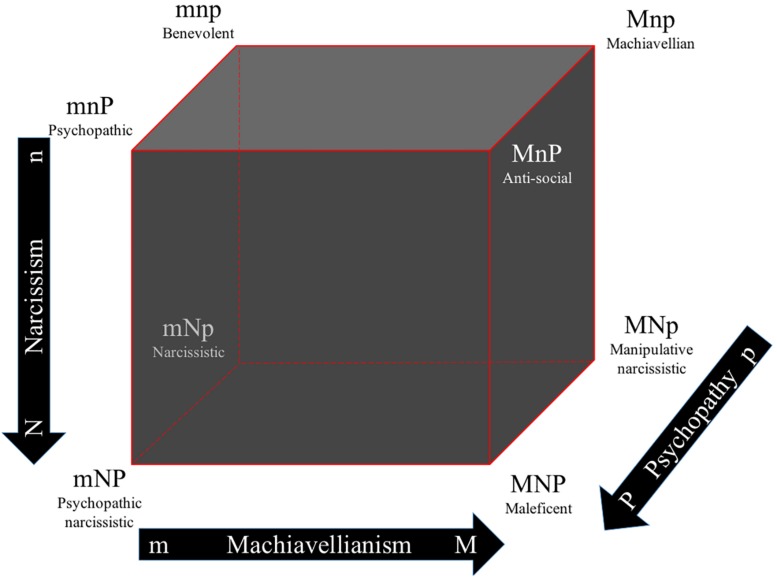
The dark cube as an analogy to Cloninger’s character cube, showing all eight possible combinations of high/low scores in Machiavellianism, narcissism, and psychopathy. Adapted with permission from C. R. Cloninger. Originally published in: Garcia and Rosenberg (2016) The dark cube: dark and light character profiles. M/m, high/low Machiavellianism; N/n, high/low narcissism; P/p, high/low psychopathy.

Peoples’ propensities to amoral behavior, manipulativeness, opportunism, selfishness, callousness, and self-centeredness are suggested to be reflected in individual differences in three dark character traits: Machiavellianism, narcissism, and psychopathy (Paulhus and Williams, 2002). At a general level, this outlook of separateness (cf. Cloninger, 2004, 2007, 2013) expressed by any of these dark traits also express uncooperativeness as one common aspect of a vicious character (e.g., Garcia and Rosenberg, 2016; Moshagen et al., 2018) and different levels of other personality tendencies (Vernon et al., 2008). At the conceptual level, individuals high on Machiavellianism are cold, manipulative, and have a sarcastic worldview (Christie and Geis, 1970; Jones and Paulhus, 2014). Individuals high on narcissism lack empathy, have fantasies of enormous power, beauty and success, have low self-esteem, and are exhibitionistic and exploitative (Raskin and Hall, 1979). In other words, they regard themselves as better, smarter, more dominant and superior than others but at the same time tend to be sensitive to criticism and with a need for constant reassurance. Individuals high on psychopathy show low empathy, low anxiety, are impulsive, and thrill seeking (Hare, 1985). Although individuals high in Machiavellianism and psychopathy can be described using the same terms (e.g., manipulative and callous), those high on psychopathy are impulsive, reckless, aggressive, and lack the same convincing social skills that individuals high on Machiavellianism display (Hawley, 2003). Individuals high on narcissism are also expected to display callousness and manipulation, but they are expected to show self-enhancement as well. Accordingly, these malevolent traits, often labeled the Dark Triad (Paulhus and Williams, 2002), are addressed as overlapping constructs that can be measured separately, since they are considered to be distinctive enough (see Persson, 2019 for another point of view). Behavioral studies, for example, show that while Machiavellianism and psychopathy predict cheating when it required an intentional lie, psychopathy predicted cheating when punishment was a serious risk and individuals high in Machiavellianism cheated under high risk, but only if they were ego depleted (Jones and Paulhus, 2017; see also Crysel et al., 2013; Jones, 2014). Hence, as for the light character traits, the dark character traits might also be seen, at least in theory, as one dynamic complex adaptive system rather than three single traits. In this line of thinking, Garcia (Garcia and Rosenberg, 2016; Garcia, 2018) suggested, analogous to Cloninger’s “light” character cube (Cloninger, 2004), the Dark Cube, which comprises the eight possible combinations of high/low scores in the three malevolent traits (see Figure 2; Garcia and Rosenberg, 2016; Garcia and Gonzàlez, 2017; Garcia, 2018; Garcia et al., 2018).

At the operationalization level, factor-analytic studies using short measures of the Dark Triad (27 items or less) have shown that narcissism and psychopathy load on the same factor (Furnham and Crump, 2005; Garcia and Rosenberg, 2016; Kajonius et al., 2016; Persson et al., 2017, 2019). On this basis, some researchers have suggested a dyad rather than a triad (e.g., Garcia and Rosenberg, 2016), and others even suggest that, at least based on the analyses of short measures, the three traits can be described well by individuals’ response to a single item measuring their tendency to exploit others (e.g., Kajonius et al., 2016). We argue that the mapping of words and their meaning to short scales’ scores might shed some light to validate if the scales target different malevolent character traits.

### Quantitative Semantics Test Theory (QuSTT)

We have argued that since psychological phenomena is expressed in natural language (e.g., psycholexical hypothesis), if reliably quantified, the mere words people use to express, for example, their personality, can be used to validate self-report scales of the construct at hand. We quantified the words that people use when asked to describe who they are with 10 words, using the Latent Semantic Analysis algorithm. The analyses were conducted in semanticexcel^3^, which is a web-based program for the analyses of quantitative semantics developed by Sverker Sikström at Lund University, Sweden (for details, see Garcia and Sikström, 2013a,b, 2014; Garcia et al., 2015; Sikström and Garcia, 2019). Here, we just present a brief overview of how semantic representations are generated, how the self-descriptive words generated by the participants are linked to this representation and then regressed on participants’ own character traits scores, and how we map the self-descriptive words to the character traits scores. This whole procedure stands as the basis of QuSTT.

#### Creating a Semantic Representation of the English Language

Semanticexcel comprises semantic representations of several languages, including English, Spanish, Swedish, etc. The representation of English used here was generated using Google N-grams^4^, which might be the largest possible available English text corpus^5^ (see also Lin et al., 2012). First, using semanticexcel, the researcher generates a matrix where rows correspond to unique single words and each column corresponds to the 5-gram context to the words in the corpus. The rows for the English corpus used here consisted of the 120,000 most frequent words, whereas the columns consisted of the contexts of the 10,000 most common words. The contexts of the words were generated from the 5-gram of Google N-grams database, that is, for each 5-gram that each word had, the context consisted of four other words. Thus, cells in this matrix represent the frequency of occurrence of a word (rows) within a context of a word (columns). For example, the word “grateful” may have a frequency *f*_1_ in the context “aiding” and a frequency *f*_2_ in the context “accidents.” In this way, every word is represented by an array of frequencies of occurrence in each related context to a word. A basic assumption is that words with similar meaning tend to occur in the same contexts (cf. Landauer and Dumais, 1997; Landauer et al., 2007; Landauer, 2008). This implies that the vectors representing similar words are expected to point in similar direction. However, to get a good semantic representation, this word-by-context sample matrix needs to be compressed to a smaller word-by-semantic dimension matrix, where this smaller matrix tends to create a more generalized semantic representation. We conducted this data compression using singular value decomposition, a widespread dimensionality-reduction technique similar to principal component analysis. The resulting matrix is called a semantic space, which describes the semantic relatedness between words. In our analysis, the resulting semantic representation consisted of 120,000 words, where each word is represented in a vector consisting of 512 dimensions. In the present study, using semanticexcel, we simply added the vectors representing each of the 10 self-descriptive words generated by the participants. Hence, each participant’s set of 10 words obtains a quantified semantic representation based on the sum of the vectors corresponding to each of the participant’s words. For a more elaborated description, see Sikström and Garcia (2019).

#### Predicting Participants’ Character Traits Scores Based on the Semantic Representation of Their Own Self-Descriptive Words

Semanticexcel uses multiple linear regressions (*Y* = *c* × *X*), with the semantic representations as input (*X*, i.e., a participants × semantic dimensions matrix), to train the regression coefficients (*c*, i.e., a vector corresponding to the weights of each semantic dimension) to predict participants’ self-reported scores in each of the personality traits (*Y*). One multiple linear regression was conducted for each trait score. An *N*-leave (where *N* is 10% of the total dataset) out-cross validation procedure is used to evaluate the results from the multiple linear regression so that the-to-be predicted data point is removed from the training set (where the coefficients of the multiple linear regression are generated) and where these coefficients are applied to make a prediction on the left-out test data point. Thus, 10 (*N*) new training and testing sets are made for cross-validation. To avoid overfitting, a subset of the dimensions in a semantic representation is used, where fitting with too many parameters in relation to the number test data points may yield poor generalization to test dataset. This subset is selected by selecting the first (*N*) dimension in semantic representation and then optimizing the number of dimensions (*N*) used by an additional 10% leave-out procedure. Furthermore, the maximum number of dimensions used is set to one half of the total number of predicted data points. In short, semanticexcel generates the predicted values by applying the regression coefficients (*c*) from the training dataset on the test dataset. To evaluate whether participants’ personality trait scores are significantly predicted by the semantic representation of the 10 generated words, the personality trait scores are simply correlated with the predicted values. A significant positive correlation (one-tailed) indicates that the semantic representation predicts the outcome variable (i.e., the participants’ score in each of the personality traits).

#### Mapping the Frequency of Self-Descriptive Words and Self-Reported Personality Traits

**FIGURE 3:**
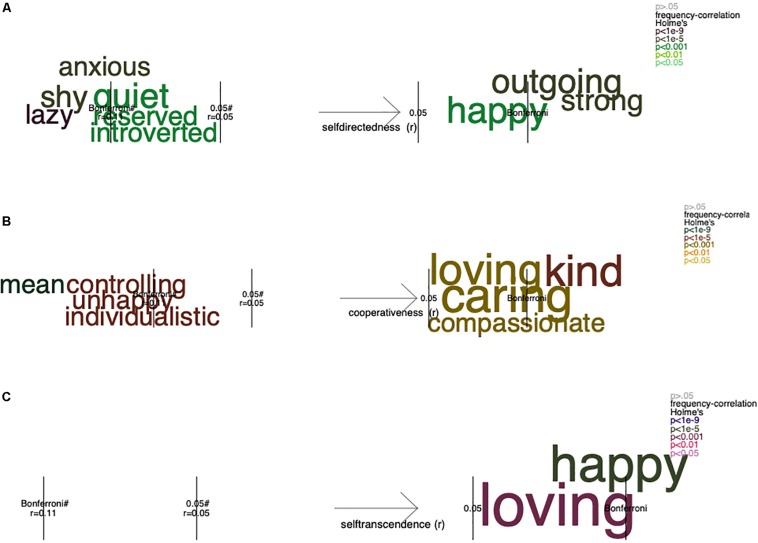
One-dimensional analysis: the frequency of the self-descriptive words that significantly correlated with participants’ scores in self-directedness **(A)**, cooperativeness **(B)**, and self-transcendence **(C)**. The figure shows, on the *x*-axis, color-coded words that significantly discriminate between the high and the low value of the scale. The area outside of the inner gray lines represents significant differences without correction for multiple comparisons (*p* = 0.05), and the areas outside of the outer gray lines represents significant values following Holm’s correction for multiple comparisons, where the number of significant words are *n* = 8 for self-directedness **(A)**, *n* = 6 for cooperativeness **(B)**, and *n* = 2 for self-transcendence **(C)**. The font size represents the frequency of occurrence of the words. The total number of unique words was 1,436, so that the percentage of unique significant words ranged from 0.14 to 0.56%. Significance testing are made by Pearson correlation to scores in each light character trait. Preliminary analyses for the results presented here were earlier published in Garcia and Sikström (2019).

Each word’s frequency was correlated to participants’ scores in each of the personality traits. To present these results, for each personality measure, we conducted one-dimensional correlations (i.e., one trait at a time) and three-dimensional correlations (i.e., interactions between high and low scores in the three character traits for each personality model). Preliminary analyses of the one-dimensional correlations presented in Figures 3, 5 were earlier published elsewhere (Garcia and Sikström, 2019).

### The Present Study

In the present study, we used quantitative semantics to validate two short personality inventories, the Short Character Inventory and the Short Dark Triad. This method allowed us to extract and represent the meaning of words based on the context in which they co-occur with other words. We expected that the quantified meaning of words that an individual use to intentionally describe herself/himself may predict her/his level in different personality traits, thus, allowing the validation of each trait measurement. We also mapped the self-presentation words to responses in each scale and also to any interaction between the traits within each personality model (i.e., light character profiles and dark character profiles).

### Ethics Statement

Ethics approval was not required at the time the research was conducted as per national regulations. The consent of the participants was obtained by virtue of survey completion after they were provided with all relevant information about the research (e.g., anonymity, possibility to withdraw at any time, etc.).

## Study 1: Light Character

### Method

#### Participants and Procedure

The participants were recruited from Mechanical Turk (MTurk)^6^. In the initial stage, we informed the participants that the survey was anonymous, voluntary, and that they could stop the survey at any time. The participants received a small compensation/reward of USD 0.50. for participating and were requested, through the Amazon system, to be residents of the United States and to have American English as their mother tongue. We added two control questions to control for automatic responses (i.e., This is a control question, please answer “neither agree or disagree”). Three out of 1,000 participants failed to respond correctly to this question; thus, the final sample comprised 997 participants (age *M* = 34.13, SD = 11.92; 363 male, 634 female).

### Instruments

#### The 10 Words Personality Inventory

This instrument was designed to request participants to freely generate words they use for self-description (Garcia and Sikström, 2015, 2019). It contains one question, asking the participants to generate 10 words that describe her/his personality (“Please describe your personality using ten words”).

#### The Short Character Inventory

C. R. Cloninger designed the Short Character Inventory for Time Magazine as a brief version of the Temperament and Character Inventory that is easy to administer for testing relationships among personality variables in large groups (Cloninger et al., 1993). We obtained permission from C. R. Cloninger to include the inventory in the present study. The inventory contains 15 items, all present in the original long version, which are rated on a five-point Likert scale (1 = definitely false, 5 = definitely true). Examples of the items are the following: “Each day I try to take another step toward my goals” (self-directedness; Cronbach’s α = 0.56), “I enjoy getting revenge on people who hurt me” (cooperativeness, reversed item, Cronbach’s α = 0.54), and “Sometimes I have felt like I was part of something with no limits or boundaries in time and space” (self-transcendence, Cronbach’s α = 0.57).

### Results and Discussion

#### Semantic Representations and Self-Reported Scores of Light Character Traits

The semantic representations of the characters created using the self-descriptive words correlated significantly with the corresponding values of the self-reported traits: self-directedness: *r* = 0.33, *p* < 0.0001; cooperativeness: *r* = 0.28, *p* < 0.0001; and self-transcendence: *r* = 0.16, *p* < 0.0001 (black cells in Table 1). The intracorrelations between the self-reported scores (dark gray cells in Table 1) and the intracorrelations between the light character traits semantic representations (light gray cells in Table 1) showed a different pattern. There were significantly higher correlations (ranging between 0.46 and 0.50) between the semantic representations of the traits compared to the correlations between the self-reported scores (ranging between 0.10 and 0.29): for the correlation between self-directedness–cooperativeness was *z* = −4.43, *p* < 0.001; for self-directedness–self-transcendence was *z* = −8.85, *p* < 0.001; for cooperativeness–self-transcendence was *z* = −8.65, *p* < 0.001. Thus, these suggest that the semantic representations may not be able to discriminate between the character traits or that the items in the scales prime participants to generate words with similar meaning. This was more accentuated for the trait of self-transcendence, where the self-reported score correlated to an almost equal degree to all three semantic representations of the three light character traits: 0.14 with the semantic representation of self-directedness; 0.18 with the semantic representation of cooperativeness; and 0.16 with the semantic representation of self-transcendence. That being said, the fact that the semantic representations were so strongly related to each other, while the self-reported scores were not, suggests that the quantification of the self-descriptive words might fail to capture the nuances targeted by the scales. Other algorithms might be necessary to allow a better validation (see among others Larsen et al., 2008; Arnulf et al., 2019).

**TABLE 1:** Correlations between the semantic representation and the self-reported scores of the light character traits.

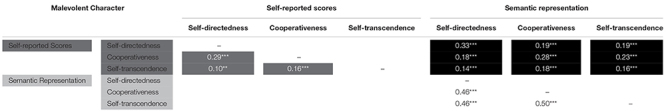

***p < 0.0001; **p < 0.001. Black cells, correlations between semantic representations and self-reported scores of light character; dark gray cells, correlations between self-reported scores of light character; light gray cells, correlations between semantic representations of light character.

#### Self-Descriptive Words and Self-Reported Scores of Light Character Traits

We conducted a correlation analysis between participants’ scores in each of the traits and the participant’s frequency of occurrence of each of the self-descriptive words (Figure 3). The 997 participants generated 1,436 words that appeared one time or more in the dataset, that is, they were “unique words.” Because the number of participants were quite large, we could find significant effect although some correlations were somewhat low (e.g., *r* = 0.11); thus, the *p* values were corrected for multiple comparisons using Holm’s correction.

The number of times that participants have generated significant words in Study 1 are found in Supplementary Table S1. In the first analysis, one-dimensional Pearson correlations, we found one word associated with both self-directedness and self-transcendence character trait scores, namely, “happy” (*n* = 180). Accordingly, Cloninger (2004, 2007, 2013) has, in a series of studies, showed that both of these character traits are associated to happiness and positive affect and emotions. Moreover, one communal word was positively associated with participants’ scores in cooperativeness and self-transcendence: “loving” (*n* = 257). The words “caring” (*n* = 320, which is the most commonly generated word, corresponding to 22% of the participants responses) and “kind” (*n* = 251), and “compassionate” (*n* = 89) were indicative only of cooperativeness. Both these traits are expressions of a person’s relation to others and the world around. Self-transcendence specifically is associated with humanistic and oceanic feelings; thus, the world “loving” might express more of a universal feeling, while “kind,” “caring,” and “compassionate” might refer to one’s relationship to others. For high levels of self-directedness, two words were indictive: “outgoing” (*n* = 150) and “strong” (*n* = 116). Both words are in line with high self-directedness (Cloninger, 2004). In addition, low self-directedness was indicated by words such as “anxious” (*n* = 63), “shy” (*n* = 123), “lazy,” “quiet” (*n* = 157), “reserved” (*n* = 77), and “introverted” (*n* = 72), hence suggesting that the self-directedness scale measures both degree of responsibility (“lazy”) and extroversion/introversion (“reserved,” “quiet,” “introverted”). Finally, low self-directedness has been found to be associated to mental illness (Cloninger, 2004), which here was indicated by the relationship to self-describing oneself as “anxious.” Indeed, other studies (e.g., De Fruyt et al., 2000) using self-reported scores have found self-directedness to correlate to neuroticism (*r* = −0.63), extraversion (*r* = 0.29), and conscientiousness (*r* = 0.45).

**FIGURE 4:**
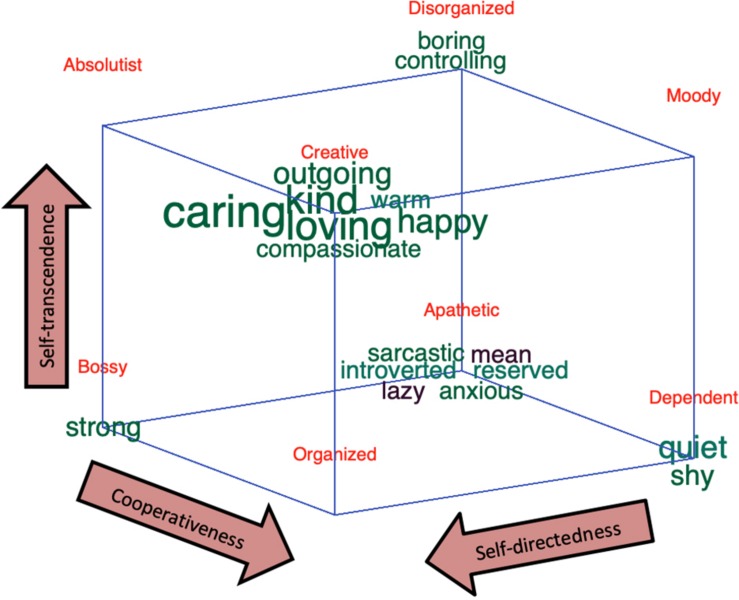
Three-dimensional analysis: the self-descriptive words mapped to the interactions between all three character traits, that is, character profiles. The analyses plot the self-descriptive words as a cube, where the corners of each cube represent words indicative of high or low values of the three character traits following Holm’s correction of multiple comparisons. Each of the eight corners of the cube represent the eight possible combinations that a word is significant for a high or low value in the three portrayed traits. For example, if a word is significant for a high value in all three traits, then it is placed in the SCT “creative” corner, whereas if it is significant for a low value of all three traits, it is placed in the sct “aphetic” corner. For details on the three axes, see the footnote in Figure 3.

We used the theorized eight profiles within the “Light” Character Cube (Cloninger, 2004) as the framework of the three-dimensional analyses (see Figure 4): SCT “creative,” SCt “organized,” ScT “absolutist,” Sct “bossy,” sCT “moody,” sCt “dependent,” scT “disorganized,” and sct “apathetic.” As expected individuals with an “apathetic” profile described themselves with words typical of a person with an immature character and high ill-being, for example, “sarcastic,” “mean,” “lazy,” and “anxious.” In contrasts, individuals with the opposite profile (i.e., “creative”) described themselves with words such as “kind,” “caring,” “loving,” “happy,” “warm,” and “compassionate.” Indeed, the combination of being highly self-directed, cooperative, and self-transcendent (i.e., “creative” character profile) facilitates a person getting in a state of calm alertness, thus allowing her/him to discover creative solutions that are adaptive for her/him, other people, and humanity at large (Cloninger et al., 2016). In contrast, people who are low in all three character traits (i.e., “apathetic” profile) feel that “life is hard, people are mean, and then you die.” (Cloninger, 2004). In other words, they feel victimized and helpless (low self-directedness and low cooperativeness) and are injudicious (low self-transcendence) and distrustful (low cooperativeness and low self-transcendence). Consequently, they experience frequent negative emotions and rare positive emotions (Cloninger, 2004). Individuals with a “bossy” profile were denoted by the word “strong.” Accordingly, Cloninger (2004) has described people with this profile as domineering (high self-directedness and low cooperativeness), logical (high self-directedness and low self-transcendence), and distrustful (low cooperativeness and low self-transcendence). They often give orders without listening to other people to gain a shared perspective because they are distrustful. Hence, using the word “strong” to describe the self makes sense in this context. Furthermore, Cloninger (2004) describes individuals with a “disorganized” profile as often being preoccupied with unrealistic fantasies and experiencing frequent distortions of reality, such as illusions and superstitions. It is unclear if the self-descriptive words associated with this profile (i.e., “boring” and “controlling”) validate this specific character combination. In contrast, the self-descriptive words associated with a “dependent” profile (“quiet” and “shy”) are a relatively good description of a person that is submissive (low self-directedness and high cooperativeness), injudicious (low self-directedness and low self-transcendence), and conventional (high cooperativeness and low self-transcendence). This creates an insecure dependent relationship in which they are not self-reliant (Cloninger, 2004).

**FIGURE 5:**
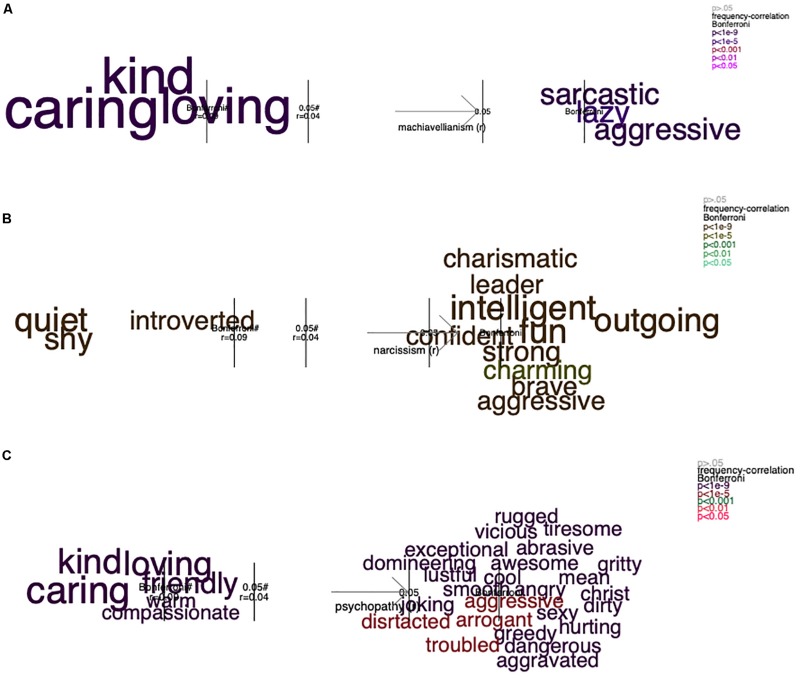
One-dimensional analysis: the frequency of the self-descriptive words that significantly correlated with participants’ scores in Machiavellianism **(A)**, narcissism **(B)**, and psychopathy **(C)**. The figure shows, on the *x*-axis, color-coded words that significantly discriminate between the high and the low values in the dark character traits. The area outside of the inner gray lines represents significant differences (*p* = 0.05), and the areas outside of the outer gray lines represents significant values following Holm’s corrections for multiple comparisons. The font size represents the frequency of occurrence of the words. The *x*-axis represents the full range of the scores in Machiavellianism **(A)**, narcissism **(B)**, and psychopathy **(C)**. For additional details, see the figure note of Figure 6. Preliminary analyses for the results presented here were earlier published in Garcia and Sikström (2019).

However, three of the profiles were not associated with any specific self-descriptive words. Thus, these specific character combinations (i.e., SCt “organized,” ScT “absolutist,” and sCT “moody”) might be less valid using the Short Character Inventory. Indeed, in recent genetic studies (Zwir et al., 2018a, b, 2019), Cloninger and colleagues have shown that the natural building blocks of personality are multifaceted profiles of the whole person, not individual traits, something that can hardly be accurately calculated using a short self-reported measure.

## Study 2: Dark Character

### Method

#### Participants and Procedure

As for Study 1, participants in Study 2 were recruited through MTurk, and we followed exactly the same protocol for the data collection. The 10 Words Personality Inventory was also used in Study 2 to ask participants to describe their personality using words. As for Study 1, we added two control questions to control for automatic responses (e.g., This is a control question, please answer “neither agree or disagree”), which eliminated 100 participants (4.04% internal dropout) from the final cohort: 2,373 participants, 845 of which were men (*M* = 33.37, SD = 11.52) and 1,527 were women (*M* = 35.44, SD = 12.78).

### Instruments

#### The 10 Words Personality Inventory

This instrument was designed to request participants to freely generate self-descriptive words (Garcia and Sikström, 2015, 2019). It contains one question, asking the participants to generate 10 words that describe her/his personality (“Please describe your personality using ten words”).

#### The Short Dark Triad

We used the Short Dark Triad (Jones and Paulhus, 2014) to measure the three dark traits: Machiavellianism, narcissism, and psychopathy. The Short Dark Triad comprises 27 items, nine per trait, that are rated on a five-point Likert scale (1 = strongly disagree to 5 = strongly agree). Examples of the items are the following: “Most people can be manipulated” (Machiavellianism; Cronbach’s α = 0.76), “People see me as a natural leader” (narcissism; Cronbach’s α = 0.76), and “Payback needs to be quick and nasty” (psychopathy; Cronbach’s α = 0.73).

### Results and Discussion

#### Semantic Representations and Self-Reported Scores of Malevolent Character Traits

The semantic representations of the malevolent characters created using the self-descriptive words correlated with the corresponding values of the self-reported dark traits: Machiavellianism: *r* = 0.19, *p* < 0.0001; narcissism: *r* = 0.35, *p* < 0.0001; and Psychopathy: *r* = 0.35, *p* < 0.0001 (see black cells in Table 2). The intracorrelations between the self-reported scores (dark gray cells in Table 2) and the intracorrelations between the dark traits semantic representations (black cells in Table 2) showed almost the same pattern: a higher correlation between Machiavellianism and psychopathy (*r* = 0.52 between self-reported scores and *r* = 0.58 for semantic representations; *z* = −2.97, *p* < 0.001), a more moderate correlation between narcissism and psychopathy (*r* = 0.39 between self-reported scores and *r* = 0.44 for semantic representations; *z* = −2.08, *p* < 0.05), and a lower correlation between Machiavellianism and narcissism (*r* = 0.34 between self-reported scores and *r* = 0.16 for semantic representations; *z* = 6.63, *p* < 0.001). Nevertheless, there were some inconsistencies. For instance, the relationship between the semantic representation of Machiavellianism and the psychopathy score (*r* = 0.23) was similar (*z* = 1.44, *p* = 0.08) to the correlation between the semantic representation of Machiavellianism and the Machiavellianism score (*r* = 0.19), that is, suggesting that Machiavellianism was less accurately assessed by either the semantic representation or the self-reported score. What is more, accordingly to recent research (e.g., Persson, 2019), Machiavellianism should be unified with psychopathy, which here is expressed by the similar correlations between the Machiavellianism self-reported score and the semantic representation of psychopathy compared to the correlation between the Machiavellianism self-reported score and the semantic representation of Machiavellianism.

**TABLE 2:** Correlations between the semantic representation and the self-reported scores of the dark traits.

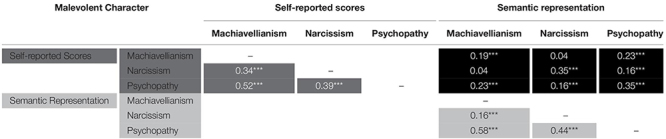

Black cells, correlations between semantic representations and self-reported scores of malevolent character; dark gray cells, correlations between self-reported scores of malevolent character; light gray cells, correlations between semantic representations of malevolent character. ***p < 0.0001.

#### Self-Descriptive Words and Self-Reported Scores of Dark Character Traits

We conducted a correlation analysis between participants’ scores in each of the traits and the participant’s frequency of occurrence of each of the self-descriptive words. The 2,373 participants generated 25,698 words, 2,367 of these appeared one time or more in the dataset; that is, they were “unique words.” In the first analysis (Figure 5), one-dimensional correlations, we found three communal words negatively associated with participants’ scores in Machiavellianism and psychopathy: “kind,” “caring,” and “loving.” In addition, only the word “aggressive” was positively related to all three dark traits. This is in line with the unification argument and past research suggesting a common, uncooperative, or disagreeable core among individuals expressing any or all of these malevolent tendencies (e.g., Paulhus and Williams, 2002; Lee and Ashton, 2005; Jakobwitz and Egan, 2006; Garcia and Rosenberg, 2016).

Furthermore, there were three words that were negatively related only to psychopathy (i.e., “friendly,” “warm,” and “compassionate”) and three words negatively related only to narcissism (“shy,” “quiet,” and “introverted”). Interestingly, all other words that were positively related to the dark traits were unique for each trait; for Machiavellianism, “sarcastic” and “lazy;” for narcissism, “charismatic,” “leader,” “intelligent,” and “confident,” “fun,” “outgoing,” “strong,” “charming,” and “brave;” and for psychopathy, “mean,” “rugged,” “vicious,” “tiresome,” “exceptional,” “abrasive,” “domineering,” “awesome,” “gritty,” “lustful,” “cool,” “mean,” “smooth,” “angry,” “Christ,” “joking,” “dirty,” “distracted,” “arrogant,” “sexy,” “greedy,” “hurting,” “troubled,” “dangerous,” and “aggravated” (see Figure 5). This finding is in line with our expectations regarding unique expressions of malevolent tendencies expressed as nuances of (un)cooperativeness—for example, the less frequent use of the word “compassionate” vs. “loving” and “kind,” which was unique for individuals high in psychopathy; the frequent use of the word “sarcastic” that was common among those high in Machiavellianism vs. the frequent use of the word “mean” that was more commonly used by individuals high in psychopathy.

The number of times that participants have generated significant words are found in Supplementary Table S2. From this table, we can see how often the participants generated words that are indicative of a trait. For example, for the trait of being high in Machiavellianism, 139 participants generated the word sarcastic, 100 lazy, and 22 aggressive. Words with positive valence tend to be generated more frequently than words with negative valence. Thus, words that were indicative of low levels of the dark traits are more commonly expressed than those that were indicative of high levels of the dark traits. For example, the words “fun” (*n* = 377), “outgoing” (*n* = 346), “sarcastic” (*n* = 135), “leader” (*n* = 47), “charismatic” (*n* = 35), and “mean” (*n* = 25) were less frequently used than “caring” (*n* = 774), “kind” (*n* = 618), “quiet” (*n* = 379), and “warm” (*n* = 156), “shy” (*n* = 315), and “introvert” (*n* = 168). Indeed, people tend to self-enhance (i.e., the desire of maximizing the positivity of self-views) and self-protect (i.e., the desire and preference for minimizing the negativity of self-views) in their self-presentations (Rosse et al., 1998; Rowatt et al., 1998) even when there is apparently no reason to appear more desirable (Tice et al., 1995; see also Amato et al., in press). However, individuals high in any of the Dark Triad traits seem to do less so, more specifically with regard to communal self-presentations. Although, we already can see in this first analysis that some words and nuances of cooperative self-presentation words discriminate between participants’ scores in each of the three dark traits, we continued with the three-dimensional analysis to control for covariance between the traits.

**FIGURE 6:**
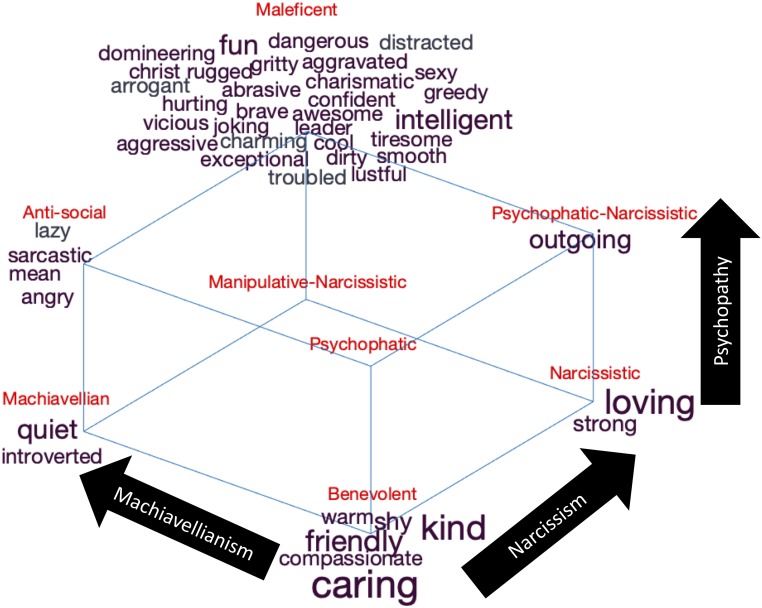
Three-dimension analysis: the self-descriptive words mapped to the interactions between all three dark character traits, that is, dark character profiles. The figure shows words where the frequency of occurrences significantly correlates with the scores on Machiavellianism (*x*-axis; 6, or 0.26% of the unique words, are significant after Holm’s correction for multiple comparisons 214 data points that are significant without correction for multiple comparisons of a total of 2,277 data points, including the comparison dataset), narcissism (*y*-axis; 13 words, or 0.57% of the unique words, are significant after Holm’s correction for multiple comparisons 225 data points that are significant without correction for multiple comparisons of a total of 2,277 data points, including the comparison dataset) or psychopathy (*z*-axis; 31 words, or 1.4%, are significant after Holm’s correction for multiple comparisons 278 data points that are significant without correction for multiple comparisons of a total of 2,277 data points). Significance testing were made by Pearson correlation to the dark traits scores. The value on the *x*-axis and the *y*-axis correlates *r* = 0.22, *p* = 0.0000. The value on the *x*-axis and the *z*-axis correlates *r* = 0.45, *p* = 0.0000. The value on the *y*-axis and the *z*-axis correlates *r* = 0.29, *p* = 0.0000. The words are plotted as word clouds on the corners of the three-dimensional Dark Cube representing these dark traits. The font size represents the frequency of occurrence of the words. The Dark Cube was adapted with permission from C. R. Cloninger, and it was originally published in Garcia and Rosenberg (2016).

We used the theorized eight profiles within the Dark Cube (Garcia and Rosenberg, 2016) as the framework of the three-dimensional analysis. The results are displayed in Figure 6 and consist of words that significantly correlated with at least one of the three dimensions, following Holm’s correction for multiple comparisons. These words were located in one of the eight corners of the cube, depending on whether they were more or less common on each of the three dimensions. Individuals with a benevolent profile (i.e., low on all three traits) used the words “warm,” “shy,” “kind,” “friendly,” “compassionate,” and “caring” more frequently in their self-presentations. This is, again, reinforcing the unification argument suggesting a common, uncooperative, or disagreeable core among individuals expressing any or all of these malevolent tendencies (e.g., Paulhus and Williams, 2002; Lee and Ashton, 2005; Jakobwitz and Egan, 2006; Garcia and Rosenberg, 2016).

Individuals high in Machiavellianism and low in both narcissism and psychopathy (i.e., Machiavellian profile) used words such as “quiet” and “introvert” less frequently. Together with the one-dimensional analysis, this suggests that individuals low in narcissism do present themselves as “quiet” and “introverted” but only if they at the same time are low in psychopathy and high in Machiavellianism. Conversely, individuals low in Machiavellianism and psychopathy but high in narcissism (i.e., narcissistic profile) used “loving” less frequently and “strong” more frequently. Indeed, highly narcissistic individuals manipulate others to gain self-validation, regardless if they hurt someone in doing so (Watson et al., 1984), which here is expressed as them presenting themselves as “strong.” In addition, low levels of narcissism seem to be associated to being “loving” only when the individual is low in the other two malevolent traits, but to being “quite” and “introvert” when the individual is high in Machiavellianism and low in psychopathy.

Individuals with psychopathic (high in psychopathy and low in the other two) or manipulative–narcissistic profiles (high in both Machiavellianism and narcissism and low in psychopathy) seem to be harder to spot by only the use of self-presentations since none of the words correlated significantly with any of these profiles, while those with a psychopathic-narcissistic profiles (high in narcissism and psychopathy and low in Machiavellianism) expressed being “outgoing,” and those individuals with an antisocial profile (high in Machiavellianism and psychopathy and low in narcissism) expressed being “lazy,” “sarcastic,” “mean,” and “angry.” Together with the one-dimensional analysis, this suggest that high Machiavellianism can be expressed by being, for example, “lazy” and “sarcastic” but only when psychopathy is high and narcissism is low. Likewise, psychopathy is expressed as being “mean” but only when Machiavellianism is high and narcissism is low. Indeed, past research suggest that individuals high in Machiavellianism and psychopathy are also low in self-discipline and that they also lack sense of duty (i.e., “lazy”) (Paulhus and Williams, 2002). Last but not the least, the Maleficent profile (i.e., high in all three dark traits) was expressed with most of the words, thus depicting a dark and malevolent character (see Figure 6).

## Conclusion

In the present set of studies, we used quantitative semantics to validate two short personality inventories, the Short Character Inventory and the Short Dark Triad. This method allowed us to extract and represent the meaning of words based on the context in which they co-occur with other words. We predicted that the quantified meaning of words that individuals use to describe themselves intentionally may predict their scores in different personality traits, thus allowing the validation of each trait measurement. We also mapped the self-presentation words to responses in each scale and also to any interaction between the traits within each personality model (i.e., light and dark character profiles).

### Limitations and Final Remarks

Despite the limitations of our data collection method through MTurk (e.g., Buhrmester et al., 2011; Goodman et al., 2013), our study showed that the traits measured by both inventories are associated to the meaning of words people use for self-description. At the general level, each self-reported score was related to the semantic representation of each respective character trait. However, participants’ self-transcendence (Study 1) and Machiavellianism scores (Study 2) demonstrated similar relationships to all three semantic representations of the character traits in their respective personality model. That being said, many of the correlations were relatively low, which might be explained by the fact that individuals were not explicitly asked to describe specific traits with their own words but their personality *per se*. Instead, the one-dimensional analyses of specific words were more informative in the validation of specific traits. Indeed, some words were indicative of both high and low levels of the character traits in each model. At the three-dimensional level, we found specific keywords that unify or that make the individuals in some profiles unique. Nevertheless, some of the profiles were not associated to any specific words. For instance, in recent genetic studies (Zwir et al., 2018a, b, 2019; Cloninger et al., 2019), Cloninger and colleagues have shown that the natural building blocks of personality are multifaceted profiles of the whole person, not individual traits. Something that can hardly be accurately calculated using short self-reported measures. Last but not the least, the measure for the light character traits is an extremely shortened version of Cloninger’s Temperament and Character Inventory, and the Dark Triad measure is far from being the best measure of malevolent character. This is certainly a problem for the measures used here (e.g., the measure for light character had Cronbach’s alphas that did not exceed 0.60). This is of course, partially, due to the low number of items.

In sum, despite being short, it seems like both inventories capture individuals’ identity as it could be expected. Nevertheless, our method also points out some shortcomings and overlaps between traits measured with these two short personality inventories. Hence, we suggest that self-descriptive words can be quantified to validate measures of psychological constructs (e.g., using self-descriptive words in natural language and QuSTT) and that this method may complement traditional methods for testing the validity of psychological measures. Finally, since it is beyond the scope of the present study, future studies need to address the fundamental question of how the mapped words might be the base of a trait description of individuals who are high and low in different character traits. For example, as our results show, is a person high in Machiavellianism best described as sarcastic, lazy, and aggressive?

“I tried to gain an idea of the number of the more conspicuous aspects of the character by counting in an appropriate dictionary the words used to express them. I examined many pages of its index here and there as samples of the whole, and estimated that it contained fully one thousand words expressive of character, each of which has a separate shade of meaning, while each shares a large part of its meaning with some of the rest (Galton, 1884, p. 181).”

## Data Availability Statement

The datasets generated for this study are available on request to the corresponding author.

## Ethics Statement

Ethics approval was not required at the time the research was conducted as per national regulations. The consent of the participants was obtained by virtue of survey completion after they were provided with all relevant information about the research.

## Author Contributions

DG, PR, KC, and SS contributed to the conception and design of the study. DG, PR, AN, and AG collected the data. SS and DG performed the statistical analysis. DG and PR wrote the first draft of the manuscript. All authors wrote the sections of the manuscript, contributed to the manuscript revision, read, and approved the submitted version.

## Conflict of Interest

The authors declare that the research was conducted in the absence of any commercial or financial relationships that could be construed as a potential conflict of interest.

## Footnotes

1 Jingle refers to two constructs with equivalent labels that really reflect different phenomena, whereas jangle refers to when one construct is given multiple names (Kelley, 1927; Block, 1995).

2 http://anthropedia.org

3 www.semanticexcel.com

4 “In the fields of computational linguistics and probability, an n-gram is a contiguous sequence of n items from a given sequence of text or speech. The items can be phonemes, syllables, letters, words, or base pairs according to the application. The n-grams typically are collected from a text or speech corpus. When the items are words, n-grams may also be called shingles” (Retrieved from http://en.wikipedia.org/wiki/N-gram).

5 https://books.google.com/ngrams

6 MTurk is an online system by Amazon.com (www.mturk.com/mturk/welcome) that provides access to a wide range of participants for research and other tasks. Each participant receives a payment for his/her work, and the amount varies depending on the size of the assignment. According to Goodman et al. (2013), 16 of America’s top 30 universities use MTurk to collect data. Rand (2011) verified that MTurk’s demographic answers are correct, and Buhrmester et al. (2011) have validated the psychometric properties of the answers in relation to data collected among undergraduate students and clinical samples.
