# Supplementary material for: Validation of Two Short Personality Inventories Using Self-Descriptions in Natural Language and Quantitative Semantics Test Theory
Source: Front Psychol. 2020 Feb 19;11:16. doi: 10.3389/fpsyg.2020.00016 (PMC7043268; doi:10.3389/fpsyg.2020.00016)
Supplement: Supplementary file 1 [file Table_1.pdf]

SUPPLEMENTARY MATERIAL

Validation of two Short Personality Inventories using Self-descriptions in Natural Language and Quantitative Semantics Test Theory

Danilo Garcia[1, 2, 3*], Patricia Rosenberg[1], Ali Al Nima[1, 2], Alexandre Granjard[1, 2], Kevin M. Cloninger[1, 4], Sverker Sikström[4*]

[1]Blekinge Center of Competence, Region Blekinge, Karlskrona, Sweden

[2]Department of Psychology, University of Gothenburg, Gothenburg, Sweden

[3]Department of Behavioral Science and Learning, Linköping University, Linköping, Sweden

[4]Anthropedia Foundation, St. Louis, Missouri, USA

[5]Department of Psychology, Lund University, Lund, Sweden

*Correspondence concerning this article should be addressed to D. Garcia, danilo.garcia@icloud.com, or S. Sikström, sverker.sikstrom@psy.lu.se.

Table S1. Frequency of significant words in Study 1

Self-descriptive word	Frequency
caring	320
loving	257
kind	251
happy	180
quiet	157
outgoing	150
shy	123
strong	116
compassionate	89
reserved	77
introverted	72
anxious	63
lazy	38
mean	11

Note. The table lists the number of times that the significant words have been produced by the participants in Study 1, and that were produced at least ten times.

Table S2. Frequency of significant words in Study 2

Self-descriptive word	Frequency
Caring	774
kind	618
loving	590
happy	435
quiet	379
fun	377
outgoing	346
shy	315
strong	248
compassionate	220
introverted	168
warm	156
anxious	144
sarcastic	139
lazy	100
leader	47
charming	43
brave	42
cool	38
charismatic	35
angry	33
mean	25
aggressive	22
sexy	17
arrogant	13
careing	11

Note. The table lists the number of times that the significant words have been produced by the participants in Study 2, and that were produced at least ten times.
